# Characterization of cold stress responses in different rapeseed ecotypes based on metabolomics and transcriptomics analyses

**DOI:** 10.7717/peerj.8704

**Published:** 2020-03-31

**Authors:** Hongju Jian, Ling Xie, Yanhua Wang, Yanru Cao, Mengyuan Wan, Dianqiu Lv, Jiana Li, Kun Lu, Xinfu Xu, Liezhao Liu

**Affiliations:** College of Agronomy and Biotechnology, Southwest University, Chongqing, China

**Keywords:** Cold stress, *Brassica napus*, Metabolome, Transcriptomics, Molecular mechanisms

## Abstract

The winter oilseed ecotype is more tolerant to low temperature than the spring ecotype. Transcriptome and metabolome analyses of leaf samples of five spring *Brassica napus* L. (*B. napus*) ecotype lines and five winter *B. napus* ecotype lines treated at 4 °C and 28 °C were performed. A total of 25,460 differentially expressed genes (DEGs) of the spring oilseed ecotype and 28,512 DEGs of the winter oilseed ecotype were identified after cold stress; there were 41 differentially expressed metabolites (DEMs) in the spring and 47 in the winter oilseed ecotypes. Moreover, more than 46.2% DEGs were commonly detected in both ecotypes, and the extent of the changes were much more pronounced in the winter than spring ecotype. By contrast, only six DEMs were detected in both the spring and winter oilseed ecotypes. Eighty-one DEMs mainly belonged to primary metabolites, including amino acids, organic acids and sugars. The large number of specific genes and metabolites emphasizes the complex regulatory mechanisms involved in the cold stress response in oilseed rape. Furthermore, these data suggest that lipid, ABA, secondary metabolism, signal transduction and transcription factors may play distinct roles in the spring and winter ecotypes in response to cold stress. Differences in gene expression and metabolite levels after cold stress treatment may have contributed to the cold tolerance of the different oilseed ecotypes.

## Introduction

As one of the most important environmental stresses, cold stress significantly affects plant growth, development and distribution, and it can be classified as chilling (<20 °C) and freezing (<0 °C) temperatures (Chinnusamy, Zhu & Zhu, 2007; Miura & Furumoto, 2013). Cell membrane stiffness, protein complex instability and photosynthesis weakness are detected under chilling stress, and much more serious injuries occur under freezing stress conditions. Many physiology and biochemistry processes can be influenced by cold stress, which is dependent on the plant species, stage of plant development, and length of the stress period. Many plant species, including *Brassica napus* L. (*B. napus*), have the ability to acclimate to the cold. However, this ability can be increased after exposure to chilling stress, and the capacity of acclimation varies greatly among species. Cold acclimation mechanisms have been fully explored and have expanded our understanding of *Arabidopsis thaliana* (*A. thaliana*), while information regarding the molecular basis of this process in *B. napus* is largely lacking despite their similar genetics.

Numerous cold-regulated genes have been identified in diverse species, including *A. thaliana* (Zhao et al., 2009), *Oryza sativa* (Chen et al., 2003; Maruyama et al., 2014; He et al., 2016; Xiaochuang et al., 2017), *Zea mays* (Bilska-Kos, Szczepanik & Sowinski, 2016; Riva-Roveda et al., 2016), *Prunus persica* (Sanhueza et al., 2015; Bustamante et al., 2016), and many other species (Khodakovskaya et al., 2006; Kosova et al., 2012; Tian et al., 2013; Zhao et al., 2014; Liu et al., 2017), using RNA sequencing (RNA-Seq) technology. The ICEI–CBF–COR genetic network response to cold acclimation is the best understood pathway in Arabidopsis (Cook et al., 2004; Chinnusamy, Zhu & Zhu, 2007). C-repeat/dehydration responsive element (DREB)-binding factors (e.g., CBF1, -2, -3, -4, -6) belonging to the AP2/ERF domain family are significantly induced in response to cold stress (Chinnusamy et al., 2003, 2007; Cook et al., 2004). In addition, other genes involved in cold stress response have also characterized using transcriptome analysis (Kaplan et al., 2004; Wu et al., 2016). Genes involved in lipid metabolism (Welti et al., 2002; Testerink & Munnik, 2005; D’Angeli & Altamura, 2016), ABA biosynthesis and signal transduction (Lee, Henderson & Zhu, 2005; Chen et al., 2014), Ca^2+^ signal transduction (Miura & Furumoto, 2013; Shi, Ding & Yang, 2015), transcription factors (TFs) (Lindemose et al., 2013) and various hormones have been detected after cold stress (Stuempfle, Nindl & Kamimori, 2010). All of these findings have expanded our understanding of molecular mechanisms in response to cold stress tolerance (Chinnusamy, Zhu & Zhu, 2007). Nevertheless, many cold stress phenotypes cannot be explained by the functions of a single gene, highlighting the need to identify additional regulatory networks and pathways, including key metabolites.

In addition to the very sophisticated gene regulatory networks, plants have evolved various complex metabolite response patterns in the low temperature stress response (Kaplan et al., 2004). Increasing evidence has shown that gene expression and biochemical pathways are altered under cold stress conditions. Sugars, lipids and amino acids, for example, have been widely reported to be involved in the cold stress response, not only for protein synthesis process but as precursors of other key metabolites in the response to cold stress (Cook et al., 2004). As key functions of the metabolite response to cold stress, many more kinds of metabolites that are responsive to cold stress must be characterized in further studies.

Transcriptome sequencing technology has matured and can provide very efficient and high-throughput screening of low temperature response genes. In previous studies, genes involved in the cell wall and membrane stabilization, calcium signaling, hormones, TFs and mitogen-activated protein kinase (MAPK) pathways, and soluble sugar and protein biosynthesis and metabolic pathways were significantly altered after cold stress using RNA-Seq technologies (Wang et al., 2013a, 2013b; Chen et al., 2014; Ren et al., 2014; Fu et al., 2016; Gong et al., 2018). Recent technological advances have made it possible to explore the molecular mechanisms underlying the response to low temperature stress using transcriptome and metabolomics techniques (Kaplan et al., 2007; Maruyama et al., 2014; Wu et al., 2016; Zhang et al., 2016). By integrating transcriptome and metabolome data, a broader and more comprehensive understanding of genes and metabolites co-regulation of low temperature stress in Arabidopsis has been performed (Kaplan et al., 2007). In rice, genes encoding proteins involved in sucrose-starch metabolism and the glyoxylate cycle were upregulated, and these changes were correlated with the accumulation of glucose, fructose and sucrose in rice after exposure to cold or dehydration, as revealed by joint metabolite, phytohormone, and gene transcript analyses (Maruyama et al., 2014). In *A. thaliana*, flavonoids are regarded as determinants of freezing tolerance and cold acclimation using 20 mutant lines based on qRT-PCR, LC–MS and GC–MS analysis (Schulz et al., 2016). Carboxylates, amino acids, carbohydrates and sugar alcohols were also made contributing to reduce freezing tolerance in the *pgm* mutant (Hoermiller et al., 2017). Joint RNA-Seq and metabolomics analyses have revealed a reactive oxygen species-dominated dynamic model response to chilling stress in two subspecies of Asian cultivated rice (*Oryza sativa*) with significant divergence in chilling tolerance (Zhang et al., 2016). Integrative analysis of transcripts and metabolites has revealed that many transcripts and metabolites are rearranged to cope with low temperature stress in *Dendrobium officinale* (Wu et al., 2016).

As the third largest oil crop in the world, rapeseed is the most important sources of vegetable oil in China. Rapeseed is the only overwintering oil crop in China. The research and breeding of cold-resistant varieties play an important role in the safety of edible oil in China. Until now, many valuable studies have been performed to examine the morphology, physiology and molecular biology of cold resistance (Burbulis et al., 2010; Chen et al., 2011), but few studies have addressed the cold resistance regulatory network mechanisms at the transcriptome and metabolome levels in rapeseed. ABA and IP3/Ca^2+^ signal transduction pathways have been identified as key actors in response to low temperature in rapeseed (Xian et al., 2017). To our knowledge, the elucidation of candidate genes or the metabolite response to low temperature in rapeseed using metabolomics and transcriptomics analysis is still very lacking.

In this study, we aim to explore the molecular mechanisms in different rapeseed ecotypes at transcriptome and metabolome levels from a wide perspective and seek clues linking genes and metabolites in response to low temperature stress. Each rapeseed ecotype (spring and winter) containing five lines were used in our study. The spring rapeseed ecotype was more sensitive to low temperature than the winter rapeseed ecotype. Many cold response genes and metabolites were detected, and common ones had greater degrees of change in the winter than in the spring rapeseed ecotypes.

## Materials and Methods

### Plant materials and cold treatments

Five winter (N5, N7, N36, N49 and N60) and five spring ecotypes (M10, M11, N240, N244 and N249) of *B. napus* were used in this study. Seeds of each line were germinated and cultivated in a petri dish under normal conditions. After 10 days, the plants were moved to plastic pots in a greenhouse with a long-day photoperiod (28 °C with 16 h light and 24 °C with 8 h dark) until the plants grew to the five- or six-leaf stage. Half plants of each line were grown under normal conditions and used as control plants. The remaining plants of each line were moved to 4 °C for 12 h with the same photoperiod. Leaves of control and treatment plants were harvested at 0 and 12 h after cold stress. All leaves were immediately frozen in liquid nitrogen and stored at −80 °C until use. Every sample consisted of leaves from more than five plants grown under the same conditions. Three biological replicates were performed, and 12 samples including control samples for winter (28W) and spring (28S) ecotypes, cold stress samples for winter (4W) and spring (4S) ecotypes were obtained and used for RNA-Seq and metabolic profiling.

### RNA extraction and RNA-Seq

Total RNA of all samples was extracted using TRIzol reagent (Invitrogen, Carlsbad, CA, USA) according to the manufacturer’s instructions. An Agilent 2100 Bioanalyzer (Agilent, Santa Clara, CA, USA) and a spectrophotometer (NanoDrop, Wilmington, DE, USA) were used to detect the integrity, quality and quantity of total RNA. Then, high-quality RNA was sent to Genedenovo Biotechnology Co., Ltd. (Guangzhou, China) and sequenced on the Illumina sequencing platform. Briefly, mRNA was extracted using dynabeads oligo (dT) and fragmented by fragmentation buffer. Double-stranded cDNAs were synthesized using reverse-transcriptase and random hexamer primers, and the cDNA fragments were purified using a QIA quick PCR extraction kit. These purified fragments were washed with EB buffer for end reparation of poly (A) addition and then ligated to sequencing adapters. Following agarose gel electrophoresis and extraction of cDNA from gels, the cDNA fragments were purified and enriched by PCR to construct the final cDNA library. The cDNA library was sequenced on the Illumina sequencing platform (Illumina HiSeq™ 2500) using the paired-end technology.

### Sequence processing and analysis

After removal of adapter and low quality sequences, clean reads were obtained and mapped to the *B*. *napus* reference genome (http://www.genoscope.cns.fr/brassicanapus/). They were then assembled using TopHat 2.0.0 and DEGseq. FPKM (fragments per kilobase of exon per million mapped fragments) was used to calculate gene expression levels, and differentially expressed genes (DEGs) were screened with Cuffdiff using the following two criteria: (i) false discovery rate *P*-value correction of <0.05 and (ii) |log2 (fold change)| > 1 (Trapnell et al., 2010). Based on blastp analysis, homologs of TFs and hormone genes in *B. napus* were screened from our previous study (Jian et al., 2018), and possible TF and plant hormone genes among these DEGs were also obtained. In addition, all sequencing data were uploaded to NCBI under accession number PRJNA530291.

### qRT-PCR analysis

Here, 1 μg of total RNA was used for cDNA synthesis using a Transcriptor First-Strand cDNA Synthesis Kit (Roche, Basel, Switzerland). qRT-PCR was performed on a Bio-Rad CFX96 Real-time System with SYBR^®^ Green PCR Supermix (CA, USA). Each reaction of 20 μL contained 10 μL of SYBR Green premix (2X) (Roche, Basel, Switzerland), 0.5 μL of each primer (10 μM), 7.0 μL of H_2_O and 2 μL of cDNA. Three biological replications with three technical replicates were used for each reaction. The following program for qPCR was used: 98 °C for 30 s, followed by 40 cycles of 98 °C for 10 s and 60 °C for 30 s. *BnActin7* and *Bn26S* gene were used as a control. All primers used in this study are shown in Table S1.

### Metabolite extraction

The metabolite extraction protocol is based on previous studies with a few modifications (Wang et al., 2018; Ying et al., 2020). The cryopreserved biomaterial samples were removed and vacuum freeze-dried. The dried sample was ground in MM 400 (Retsch, Haan, Germany) at 30 Hz for 1.5 min, 100 mg of the powder was weighed, and 1.0 ml 70% methanol containing 0.1 mg/l lidocaine was used as an internal standard for overnight extraction at 4 °C. After extraction, the sample was centrifuged at 10,000×*g* for 10 min, the supernatant was absorbed, and the sample was filtered with a microporous membrane (0.22-μm pore size), and stored in the injection bottle for LC–MS analysis. The quality control (QC) sample was prepared by mixing the sample extracts and used to analyze the reproducibility of the samples under the same treatment method.

### Metabolite detection

The data acquisition instrument system mainly included ultra-high performance liquid chromatography (Ultra Performance Liquid Chromatography, UPLC) (Shim-pack UFLC SHIMADZU CBM20A, http://www.shimadzu.com.cn/) and tandem mass spectrometry (Tandem mass spectrometry, MS/MS) (Applied Biosystems 4500 QTRAP, http://www.appliedbiosystems.com.cn/). Briefly, 5 μL of each sample was injected onto a Waters ACQUITY UPLC HSS T3 C18 column (2.1 mm × 100 mm, 1.8 μm) operating at 40 °C and flow rate of 0.4 mL/min. The mobile phases used were acidified water (0.04% acetic acid) (Phase A) and acidified acetonitrile (0.04 % acetic acid) (Phase B). Compounds were separated using the following gradient: 95:5 Phase A/Phase B at 0 min; 5:95 Phase A/Phase B at 11.0 min; 5:95 Phase A/Phase B at 12.0 min; 95:5 Phase A/Phase B at 12.1 min; 95:5 Phase A/Phase B at 15.0 min (Chen et al., 2013). The effluent was connected to an ESI-triple quadrupole-linear ion trap (QTRAP)-MS.

Mass spectrometry information was measured on API 4500 QTRAP LC/MS/MS system both in positive and negative mode, the main parameters of the linear ion trap and triple quadruple rod were as follows: electrospray ion source (electrospray ionization, ESI) temperature 550 °C, mass spectrometry voltage 5.5 kV, curtain gas (curtain gas, CUR) 25 psi. The collision-induced ionization (collision-activated dissociation, CD) parameter was set to high. In the qualitative analysis of the metabolite, the isotopic signals, repetitive signals containing K^+^ ions, Na^+^ ions and NH4^+^ ions were removed. Metabolites were analyzed with reference to internal database, MassBank (http://www.massbank.jp/), KNAPSAcK (http://kanaya.naist.jp/KNApSAcK/), HMDB (http://www.hmdb.ca/) (Wishart et al., 2013), MoTo DB (https://omictools.com/moto-db-tool) and METLIN (http://metlin.scripps.edu/index.php) (Zhu et al., 2013), as well as other available mass spectrometry public databases (Chen et al., 2013). In the triple quad (QQQ), each ion pair was scanned and detected according to the optimized declustering voltage (declustering potential, DP) and collision energy (collision energy, CE). The m/z range was set between 50 and 1,000. After mass spectrometry analysis, the original data in wiff format could be opened and browsed using Analyst 1.6.1, which could be used for qualitative and relative quantitative analysis. Cinematographic peak areas were determined and corrected to ensure the accuracy of the metabolite relative quantitative analysis.

### Multivariate statistical analysis

Principal component analysis (PCA) carried out by SPSS for Windows (Version 18.0; SPSS Inc., Chicago, IL, USA), partial least squares discriminant analysis (PLS-DA) and orthogonal partial least-squares discriminant analysis (OPLS-DA) were performed by R software package (http://bioconductor.org/packages/release/bioc/html/ropls.html) with data from 12 samples to observe differences in metabolic composition between two *B. napus* ecotypes after cold stress. The loading plot helped to identify the metabolites that contributed the most to the changes in metabolite patterns between the comparison groups. The variables far from the origin in the transverse coordinate direction contributed more to the distinction between the two groups of samples. In addition, the variables in a certain position of the load graph often provided an important contribution to the samples in the same position on the score graph. We combined the multivariate statistical analysis of the VIP value of OPLS-DA and the univariate statistical analysis of the *T* test *P* value to screen significantly different metabolites among different comparison groups. The thresholds used to determine a significant difference were VIP ≥ 1 and *T*-test *P* < 0.05.

## Results

### Transcriptome analysis for winter and spring ecotypes of *B. napus* in response to cold stress

To detect transcriptome differences in response to cold treatment between different oilseed ecotypes, we performed an RNA-Seq analysis of each five winter and spring ecotypes of *B. napus* using Illumina-based 2 × 150-bp paired-end read sequencing. In total, 598,775,190 clean reads with 587,373,972 high-quality reads were obtained from 12 libraries (Table 1). In our study, all high-quality reads were mapped to the assembled *B. napus* genome of “Darmor-bzh” (Chalhoub et al., 2014) after mapping to Ribosome. Finally, more than 80.83–82.23% of the clean reads in 12 libraries could be mapped on the reference genome (Table S2).

**Table 1:** Basic statistics of RNA-seq reads obtained from 12 libraries.

Sample	Clean data (bp)	Clean reads num	HQ clean data (bp)	HQ clean reads num (%)	Adapter (%)	Low quality (%)	poly A (%)	*N* (%)
28S1	7,248,282,600	48,321,884	7,036,617,191	47,491,286 (98.28)	301,530 (0.62)	524,164 (1.08)	11 (0)	2,441 (0.01)
28S2	6,382,584,900	42,550,566	6,163,481,119	41,679,946 (97.95)	240,588 (0.57)	629,556 (1.48)	8 (0)	230 (0.00)
28S3	6,636,738,600	44,244,924	6,417,371,237	43,394,204 (98.08)	265,830 (0.60)	584,444 (1.32)	3 (0)	220 (0.00)
4S1	6,222,662,700	41,484,418	6,008,313,279	40,646,634 (97.98)	266,786 (0.64)	570,552 (1.38)	7 (0)	216 (0.00)
4S2	7,840,323,600	52,268,824	7,581,545,439	51,217,238 (97.99)	305,172 (0.58)	730,956 (1.40)	15 (0)	7,714 (0.03)
4S3	7,932,055,800	52,880,372	7,683,042,395	51,881,556 (98.11)	310,230 (0.59)	672,336 (1.27)	14 (0)	8,111 (0.03)
28W1	7,404,752,400	49,365,016	7,180,601,844	48,458,954 (98.16)	283,500 (0.57)	607,390 (1.23)	10 (0)	7,576 (0.03)
28W2	7,129,653,000	47,531,020	6,897,653,432	46,579,764 (98.00)	270,716 (0.57)	666,158 (1.4)	19 (0)	7,172 (0.03)
28W3	8,087,136,900	53,914,246	7,843,866,798	52,953,200 (98.22)	301,946 (0.56)	642,532 (1.19)	13 (0)	8,271 (0.03)
4W1	9,033,641,700	60,224,278	8,752,058,406	59,093,420 (98.12)	386,554 (0.64)	726,226 (1.21)	8 (0)	9,031 (0.03)
4W2	8,177,355,000	54,515,700	7,920,965,399	53,481,182 (98.1)	339,772 (0.62)	678,208 (1.24)	2 (0)	8,267 (0.03)
4W3	7,721,091,300	51,473,942	7,481,296,627	50,496,588 (98.1)	333,700 (0.65)	627,856 (1.22)	7 (0)	7,892 (0.03)

**Note:**

28S1, 28S2 and 28S3 were three replications for 28S; 4S1 4S2 and 4S3 were three replications for 4S; 28W1, 28W2 and 28W3 were three replications for 28W; 4W1, 4W2 and 4W3 were three replications for 4W.

**Figure 1:**
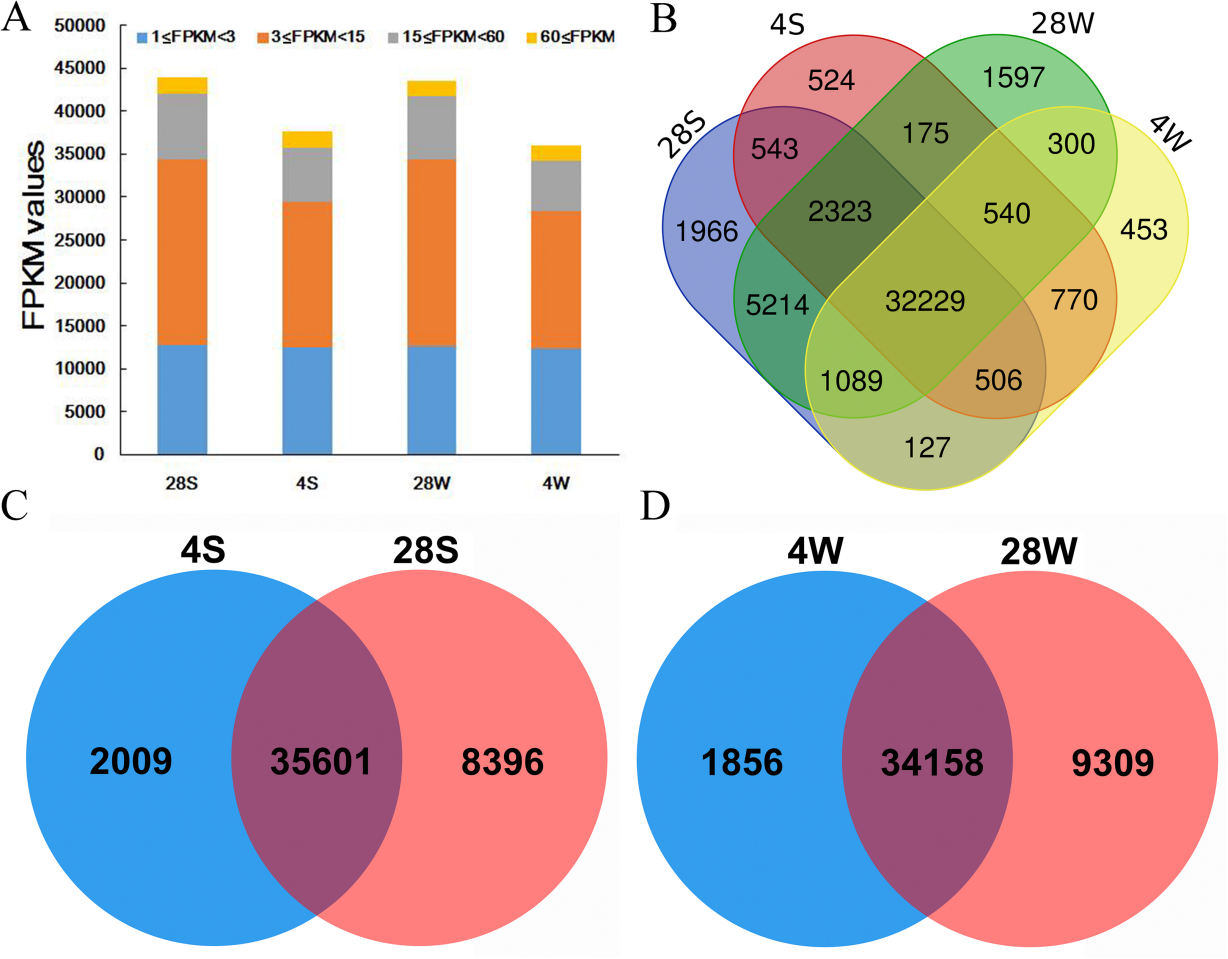
Expression statistics of winter and spring *B. napus* ecotypes in normal and cold stress conditions. (A) Statistical analysis of gene expression identified in the four libraries. (B) Venn diagram of genes detected in the four libraries. (C) Venn diagram of genes detected in the spring *B. napus* ecotype between cold stress and normal conditions. (D) Venn diagram of genes detected in the winter *B. napus* ecotype between cold stress and normal conditions.

In total, 36,014 (4W), 43,467 (28W), 37,610 (4S) and 43,997 (28S) genes were detected in our study with an FPKM value ≥1. Moreover, almost half (44.2–49.9%) of the genes in the four libraries showed moderate expression levels (3 < FPKM < 15), while only a small percentage (~5%) of the genes expressed at high levels (FPKM > 60) (Fig. 1A; Table S3). In addition, more than 66.6% (32,229/48,356) of the genes were detected in all four libraries, with 35,601 genes commonly detected in the 28S and 4S comparison, and 34,158 genes commonly detected in the 28W and 4W comparison, respectively (Figs. 1B–1D; Table S3). Furthermore, high Pearson correlations were detected among biological replicates (Fig. S1).

### Identification of DEGs in response to cold stress

**Figure 2:**
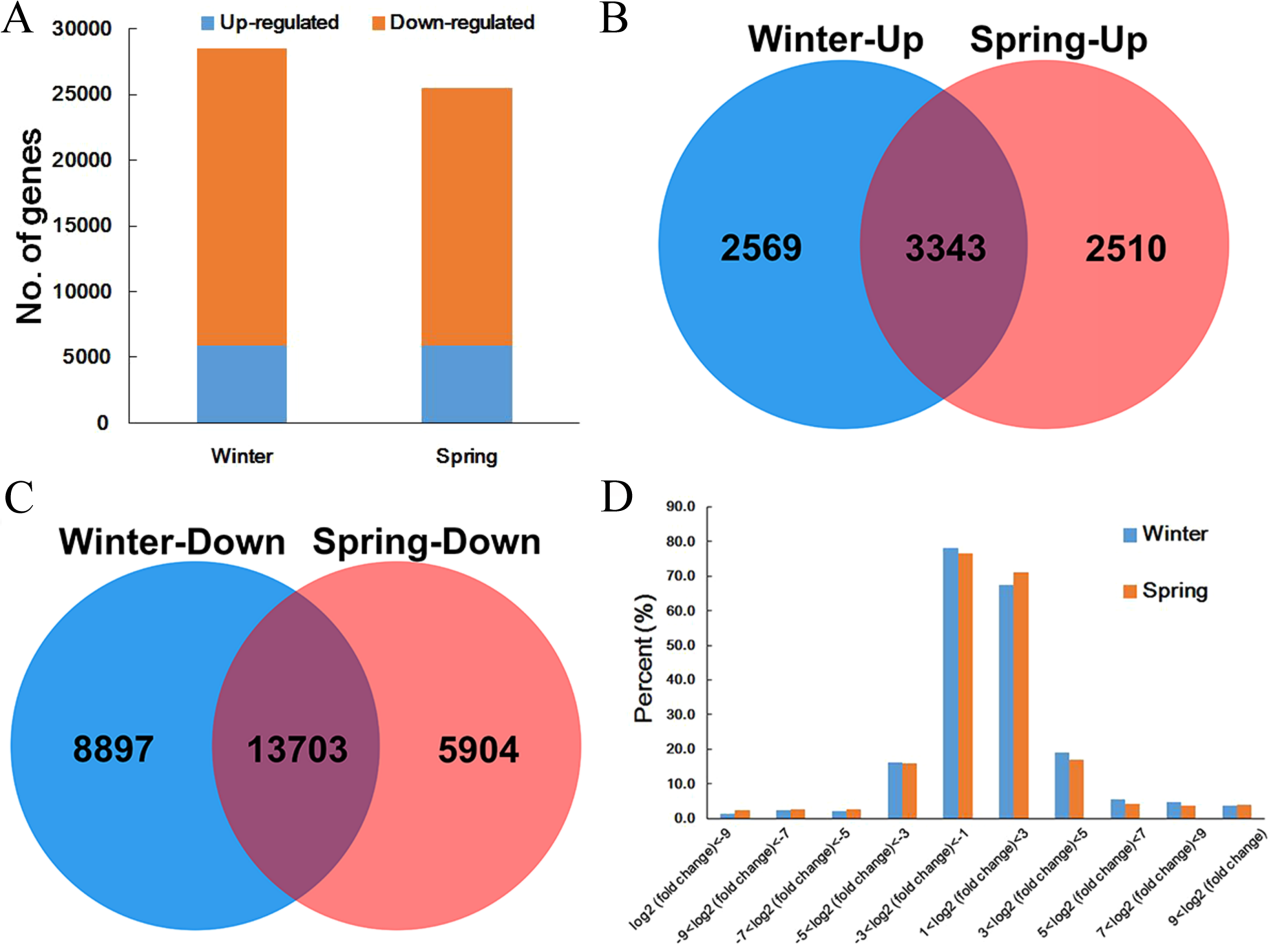
DEGs identified in the two *B. napus* ecotypes after cold stress. (A) Statistical analysis of DEGs identified in winter and spring *B. napus* ecotypes after cold stress. (B) Venn diagram of upregulated gene between spring and winter *B. napus* ecotypes after cold stress. (C) Venn diagram of downregulated gene between spring and winter *B. napus* ecotypes after cold stress. (D) Fold change statistics of DEGs identified in spring and winter *B. napus* ecotypes after cold stress.

In this study, 25,461 DEGs with 5,853 upregulated and 19,607 downregulated genes between the control and cold-treated spring ecotypes were detected. For winter ecotypes, there were 5,912 upregulated and 22,600 downregulated DEGs (Fig. 2A; Table S4). Interestingly, among the upregulated genes, 3,343 were commonly detected in two ecotypes, and 2,569 and 2,510 genes were specific to winter and spring ecotypes, respectively (Fig. 2B; Table S4). For downregulated genes, 13,703 genes were common in both winter and spring ecotypes, and 8,897 and 5,904 genes were specific to winter and spring ecotypes (Fig. 2C; Table S4). In addition, most of the DEGs (67.4% and 71.2% upregulated genes, and 78.2% and 76.5% downregulated genes in winter and spring ecotypes, respectively) had a |fold change| ≤ 8 and <4% DEGs had a |fold change| ≥ 1,024 (Fig. 2D; Table S4).

**Figure 3:**
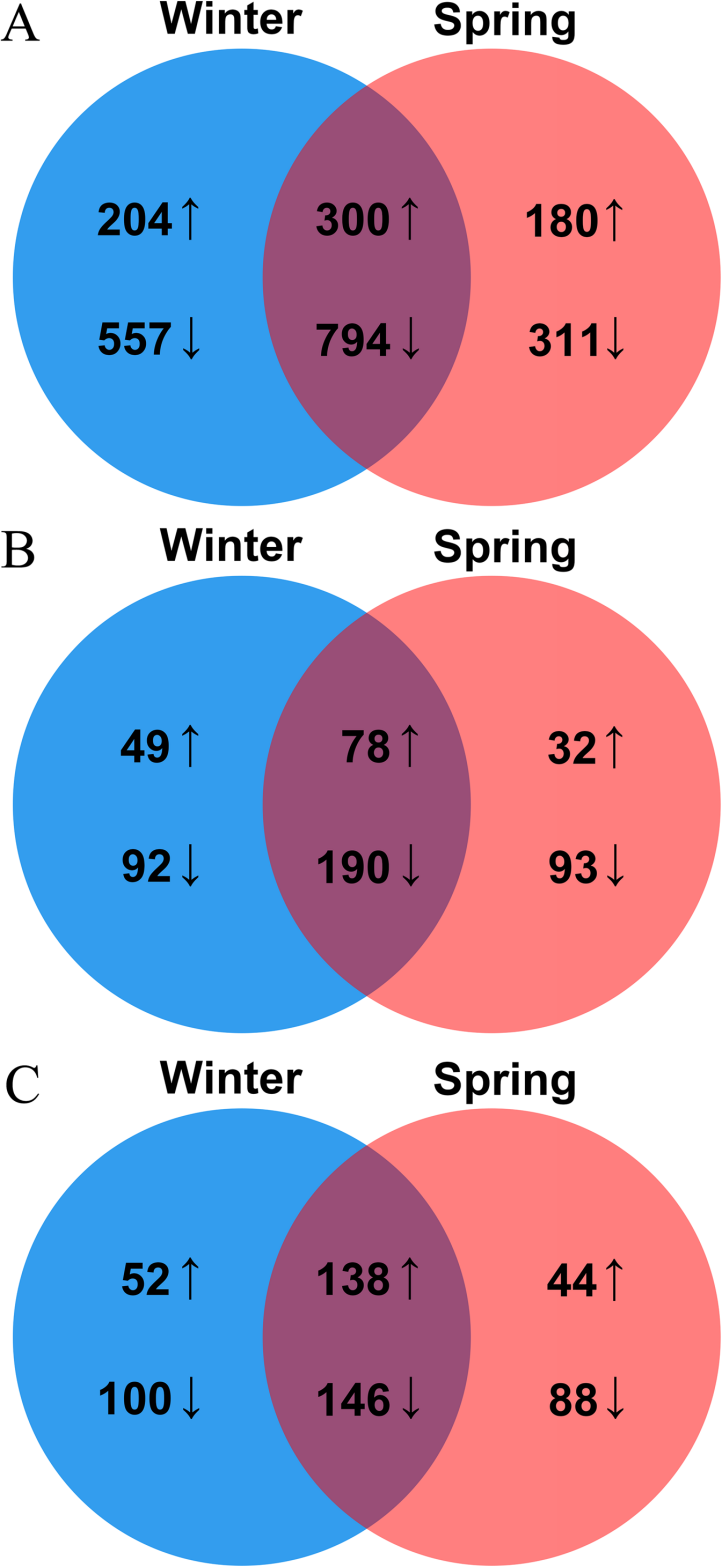
Venn diagram of DEGs encoding TFs (A), hormone biosynthesis and signal transductions (B) and temperature responsive proteins (C) between winter and spring *B. napus* ecotypes after cold stress.

As critical regulators, TFs make great contributions in response to various (a) biotic stresses in plants. In this study, 2,346 TFs genes were identified as DEGs in two ecotypes, with 1,855 and 1,585 genes in the winter and spring ecotypes, respectively. Among them, 1,094 genes including 300 upregulated and 794 downregulated genes, were commonly detected in both two ecotypes, of which 761 included 204 upregulated and 557 downregulated genes and 491 included 180 upregulated and 311 downregulated genes in the winter and spring ecotypes, respectively (Fig. 3A). These 2,346 TF genes were unevenly distributed in 53 TF families, of which the top four families, namely *bHLH*, *ERF*, *MYB* and *WRKY*, made up the majority in all groups. Interestingly, most of the *bHLH* family members were downregulated in both two ecotypes after cold stress (Table S5).

Plant hormones are not only involved in plant development, but they also have crucial functions in abiotic stresses. In total, 434 genes, including 159 upregulated and 275 downregulated, involved in hormone metabolism or signal transduction process were identified. In addition, 409 genes, including 127 upregulated and 282 downregulated, and 393 genes, including 110 upregulated and 283 downregulated, were screened in winter and spring ecotypes after cold stress, respectively (Fig. 3B). Of them, 268 genes, including 78 up- and 190 downregulated, were commonly detected in both ecotypes after cold stress (Fig. 3B). These hormone genes, ABA, auxin and ET genes made up the majority (Table S6).

In Arabidopsis, 320 genes were identified as key factors involved in low or high temperature stresses. In this study, 568 (234 upregulated and 334 downregulated) genes in total and 284 (138 upregulated and 146 downregulated) genes in common were detected in two ecotypes of *B. napus* after cold stress. In the winter ecotype, 436 genes, including 190 upregulated and 246 downregulated, and 416 genes, including 182 upregulated and 234 downregulated, were screened in winter and spring ecotypes, respectively (Fig. 3C). Among them, key genes encoded calcineurin B-like protein 1 (CBL1), cold regulated 15b (COR15B), cold regulated 15a (COR15A), cold regulated 413 plasma membrane 1 (COR413-PM1), C-repeat/DRE binding factor 1 (CBF1), CBF2, CBF4, EARLY RESPONSE TO DEHYDRATION 14 (ERD14), ERD10, ERD7, LOW-TEMPERATURE-INDUCED 65 (LTI65) and LTI41, were upregulated in both ecotypes of *B. napus* after cold stress (Table S7). Genes encoding beta-6 tubulin (TUB6), cold acclimation protein WCOR413 family, cold shock domain protein 3 (CSP3), COR413-PM2, CPL1, ERD5, ERD6, HIGH EXPRESSION OF OSMOTICALLY RESPONSIVE GENES 1 (HOS1), INDUCER OF CBF EXPRESSION 1 (ICE1), MYB15 and WRKY33 were downregulated in both ecotypes of *B. napus* after cold stress (Table S7).

**Figure 4:**
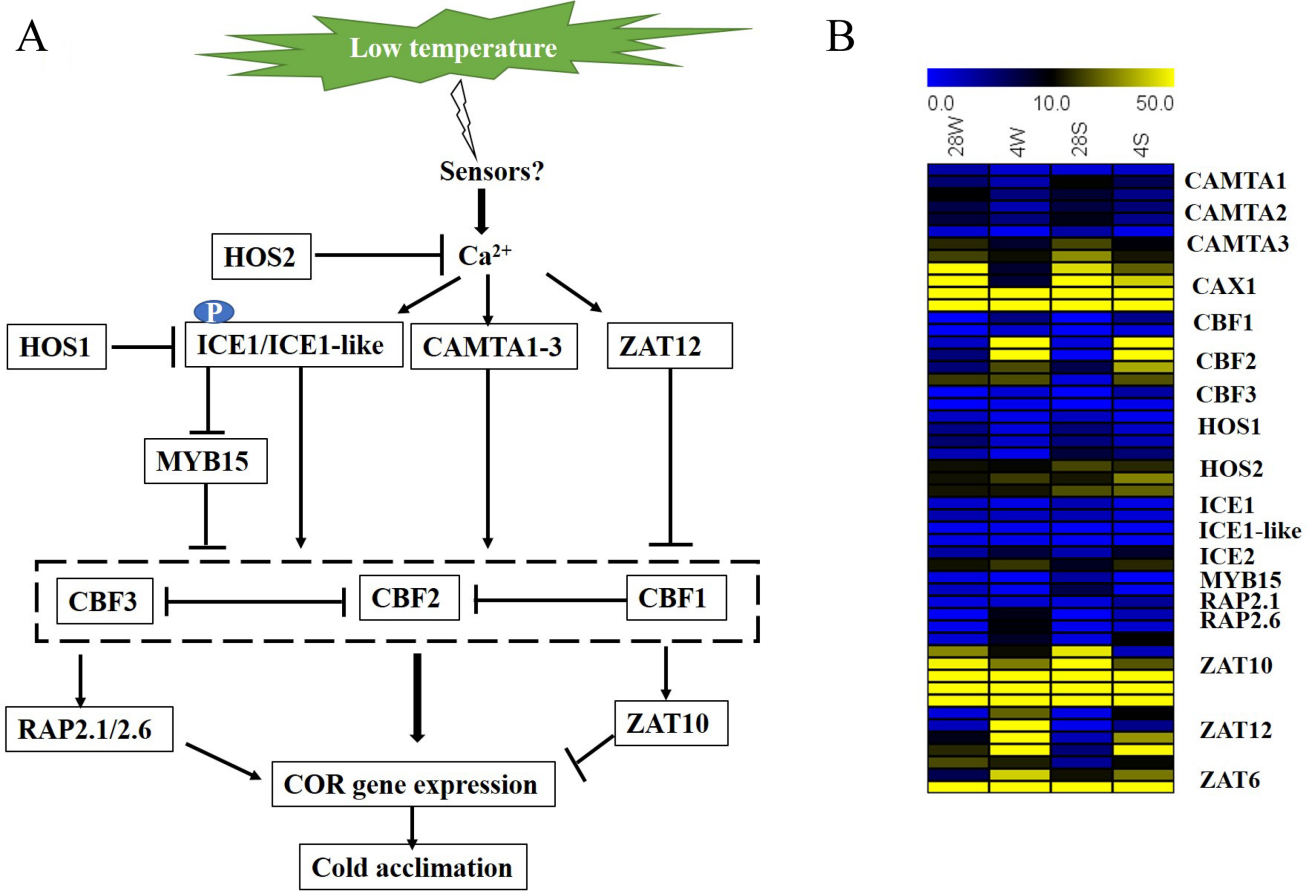
Regulation and expression of temperature-related genes in two *B. napus* ecotypes under cold stress. **(**A) Regulation model of temperature-related genes under cold stress. (B) Expression patterns (FPKM) of temperature-related genes in the two *B. napus* ecotypes under cold stress.

**Figure 5:**
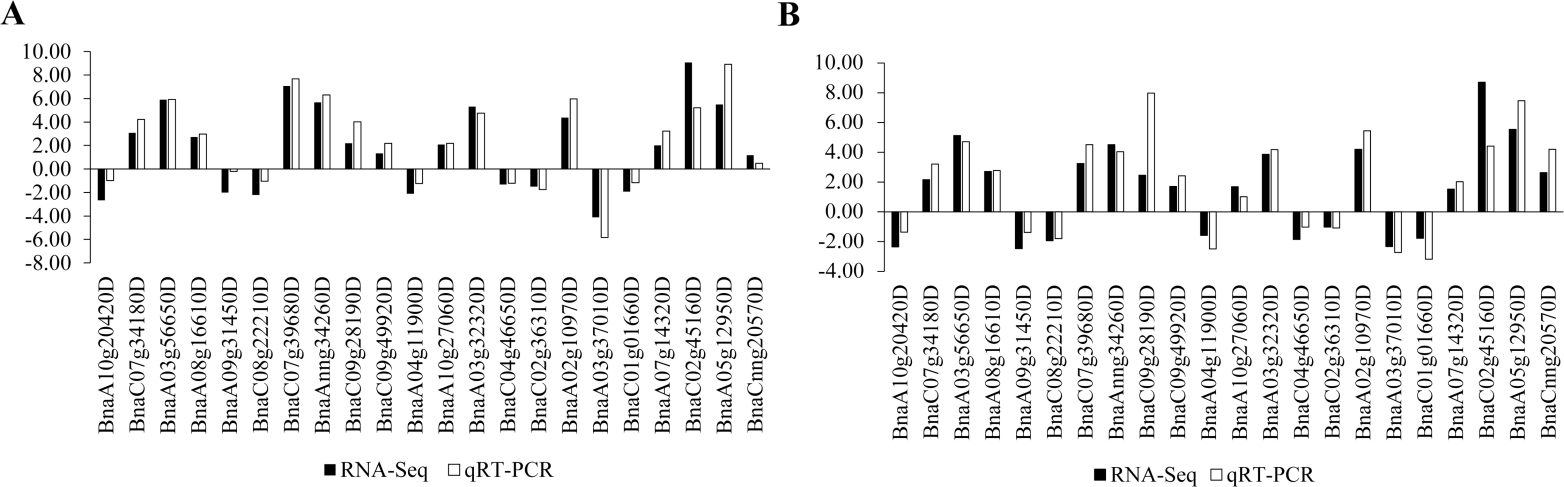
Twenty-two DEGs involved in temperature stress response were confirmed using qRT-PCR technology. (A) Confirmation of expression patterns in the winter *B. napus* ecotype after cold stress. (B) Confirmation of expression patterns in the spring *B. napus* ecotype after cold stress.

When plants perceive a low external temperature, they transmit the signal from the cell membrane to the cell, which causes a change in the calcium ion concentration on the cell membrane and triggers a phosphorylation cascade of kinases. The calcium concentration increased to promote the upregulation of ICE1 or ICE1-like, CAMTA1-3 and ZAT12, which directly or indirectly promoted the upregulation of CBFs, followed by adaption to low temperature stress through RAP2.1, 2.6 and ZAT10 (Fig. 4A). In this process, the two ecotypes of rapeseed displayed similar expression patterns, but the degree of change was slightly different (Fig. 4B). Several of them were confirmed by qRT-PCR analysis (Fig. 5). High correlation coefficients were detected between RNA-Seq and qRT-PCR technologies, suggesting that the RNA-Seq data were reliable.

### Functional analysis of DEGs involved in the response to cold stress

To classify the functions of the DEGs in the two ecotypes after cold stress, GO was used according to biological process, cellular component and molecular function (Fig. S2). Among all DEG comparisons, “metabolic process” and “cell process”, “cell part” and “cell” and “catalytic activity” and “binding” were dominant in “biological process”, “cellular component” and “molecular function”, respectively.

**Figure 6:**
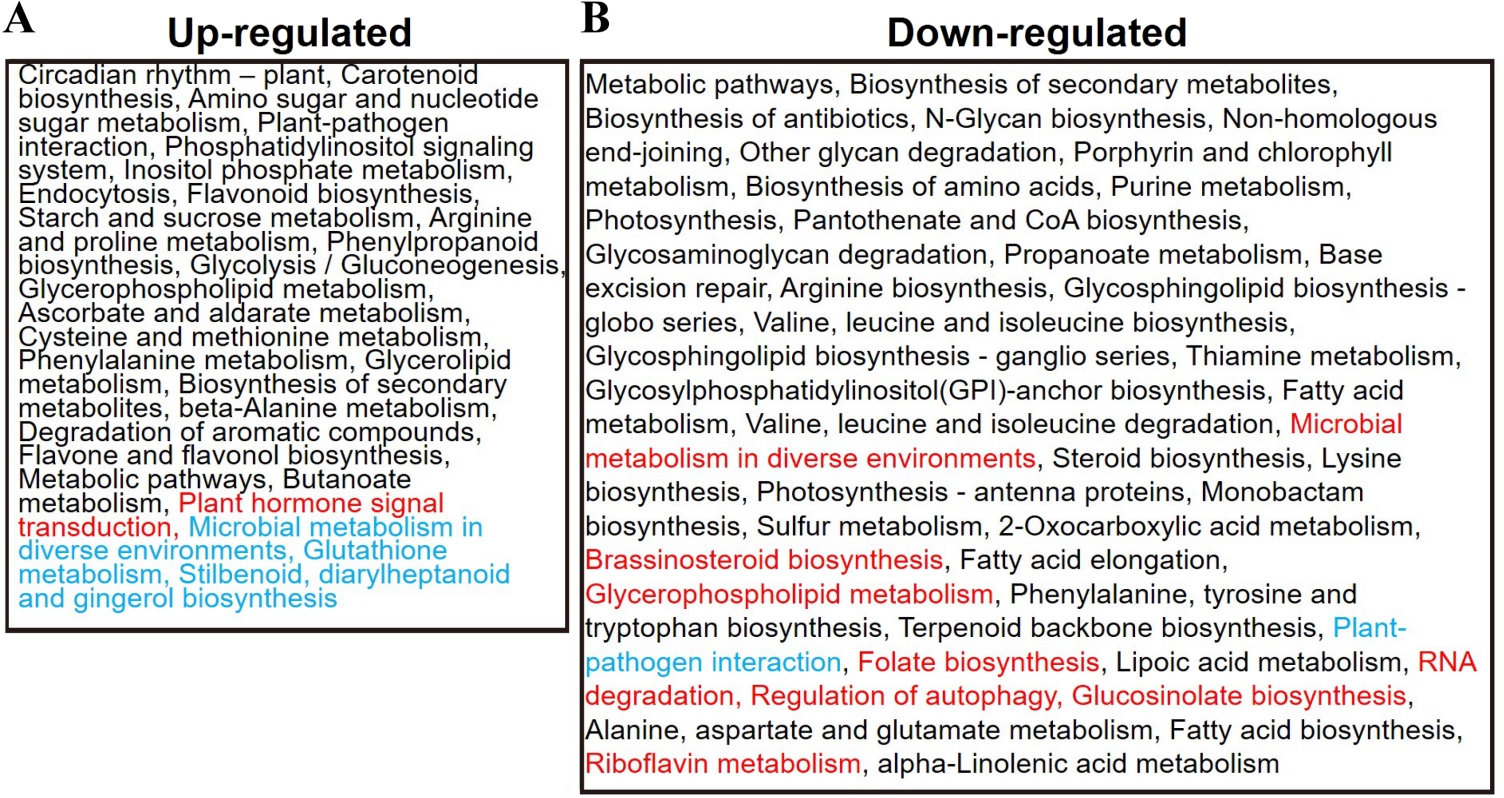
Summary of functional categories that were significantly enriched within up- and downregulated genes in the winter and spring *B. napus* ecotypes. The red font denotes categories that were only detected in the winter *B. napus* ecotype, and the blue font indicates categories were only detected in the spring *B. napus* ecotype. (A) Functional categories of up-regulated DEGs. (B) Functional categories of down-regulated DEGs.

In addition, DEGs identified in the two ecotypes after cold stress were used for KEGG pathway analysis to explore their potential functions. In total, 27 and 44 significantly enriched pathways were detected among the up- and downregulated genes, respectively (Fig. 6; Table S8). The upregulated genes that were commonly detected in the two ecotypes were mainly involved in primary metabolism, including amino acid metabolism (arginine, proline, phenylalanine, cysteine and methionine metabolism), carbohydrate metabolism (amino sugar and nucleotide sugar, inositol phosphate, starch and sucrose, glycolysis/gluconeogenesis, ascorbate and aldarate and butanoate metabolism) lipid metabolism (glycerophospholipid and glycerolipid metabolism) and secondary metabolism (flavonoid biosynthesis, phenylpropanoid biosynthesis, flavone and flavonol biosynthesis and carotenoid biosynthesis), suggesting that this reprograming of primary and secondary metabolism might represent a response to cold stress (Fig. 6; Table S8). In addition, we observed signal transduction (phosphatidylinositol signaling system), transport and catabolism (endocytosis) and environmental adaptation (circadian rhythm—plant and plant–pathogen interaction). Moreover, plant hormone signal transduction was only detected in the winter *B. napus* ecotype and glutathione metabolism and stilbenoid, diarylheptanoid and gingerol biosynthesis were only detected in the spring ecotype (Fig. 6; Table S8), suggesting that hormone signaling and secondary metabolism were involved in the cold stress response in different *B. napus* ecotypes.

Conversely, among the downregulated genes commonly detected in the two ecotypes were pathways involved in amino acid metabolism (arginine biosynthesis, valine, leucine and isoleucine biosynthesis, lysine biosynthesis and valine, leucine and isoleucine degradation, phenylalanine, tyrosine and tryptophan biosynthesis and alanine, aspartate and glutamate metabolism), carbohydrate metabolism (propanoate metabolism), lipid metabolism (steroid biosynthesis, fatty acid elongation, fatty acid biosynthesis and alpha-linolenic acid metabolism), nucleotide metabolism (purine metabolism) and glycan biosynthesis and metabolism (Fig. 6; Table S8). In addition, energy metabolism genes (photosynthesis, sulfur metabolism and photosynthesis-antenna proteins) also seemed to be downregulated. Plant–pathogen interaction pathways were only detected in the spring *B. napus* ecotype after cold stress (Fig. 6; Table S8). In the winter *B. napus* ecotype, eight pathways were also detected: glucosinolate biosynthesis, RNA degradation, microbial metabolism in diverse environments, glycerophospholipid metabolism, folate biosynthesis, riboflavin metabolism, brassinosteroid biosynthesis and regulation of autophagy (Fig. 6; Table S8), suggesting that different pathways were involved in different ecotypes after cold stress.

**Figure 7:**
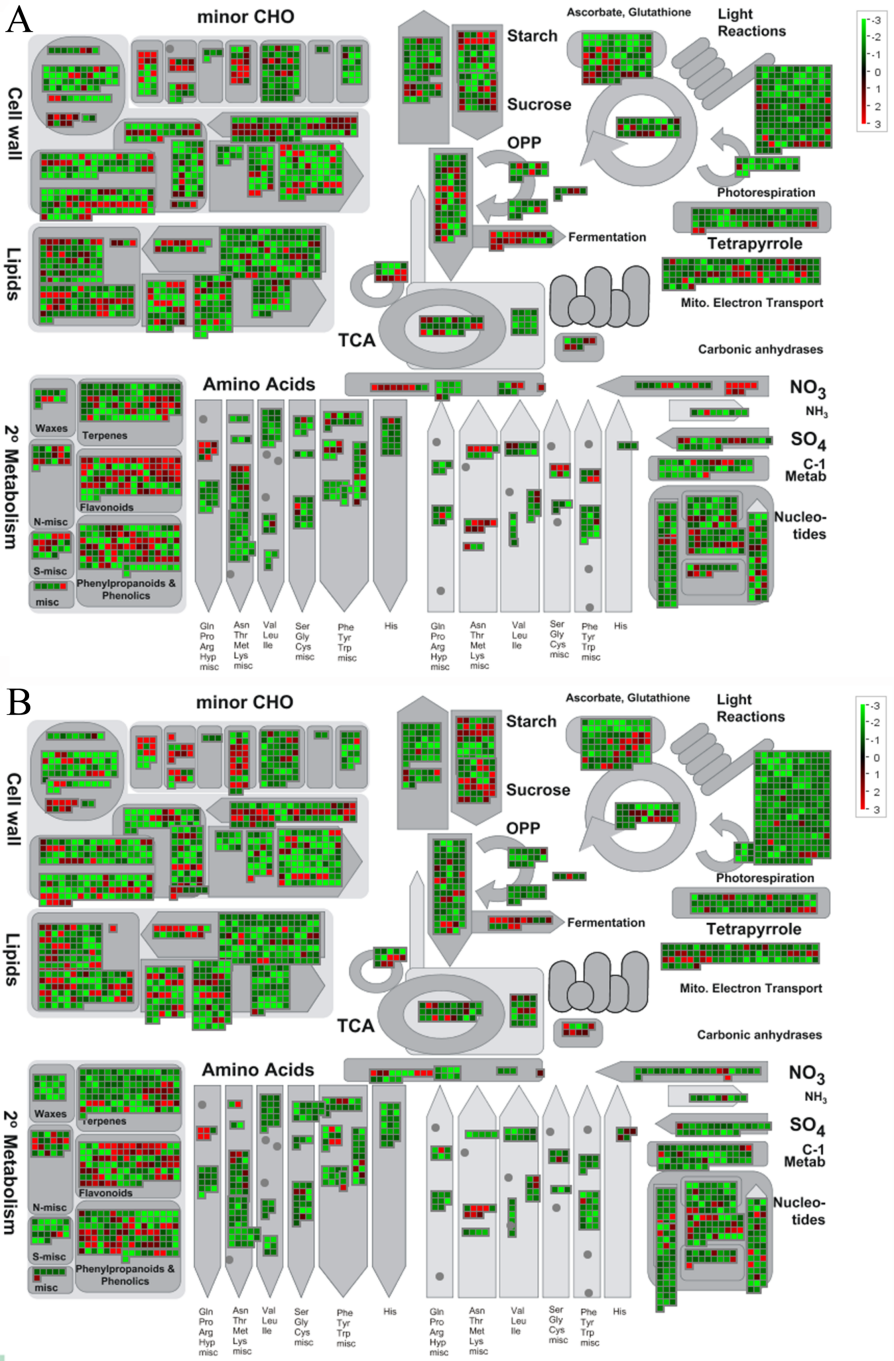
Overview of DEGs involved in different metabolic processes. The images were drawn using MapMan and show different functional categories of DEGs identified in this study. Each box depicts an individual gene. Red and green represent up-DEGs and down-DEGs, respectively. The scale bar represents fold change values. (A) DEGs identified in the spring *B. napus* ecotype after cold stress. (B) DEGs identified in winter *B. napus* ecotype after cold stress.

To further explore the molecular mechanisms underlying the response to cold stress in the two *B. napus* ecotypes, the software MapMan was used to classify the DEGs involved in the metabolic pathways (Fig. 7). As shown in Fig. 7, genes involved in photosynthesis and circadian rhythm were mostly suppressed, while cell wall, lipids, and biosynthesis of secondary metabolites were mostly activated. All DEGs involved in different metabolic pathways are summarized in Tables S9 and S10.

### Metabolome analyses

**Figure 8:**
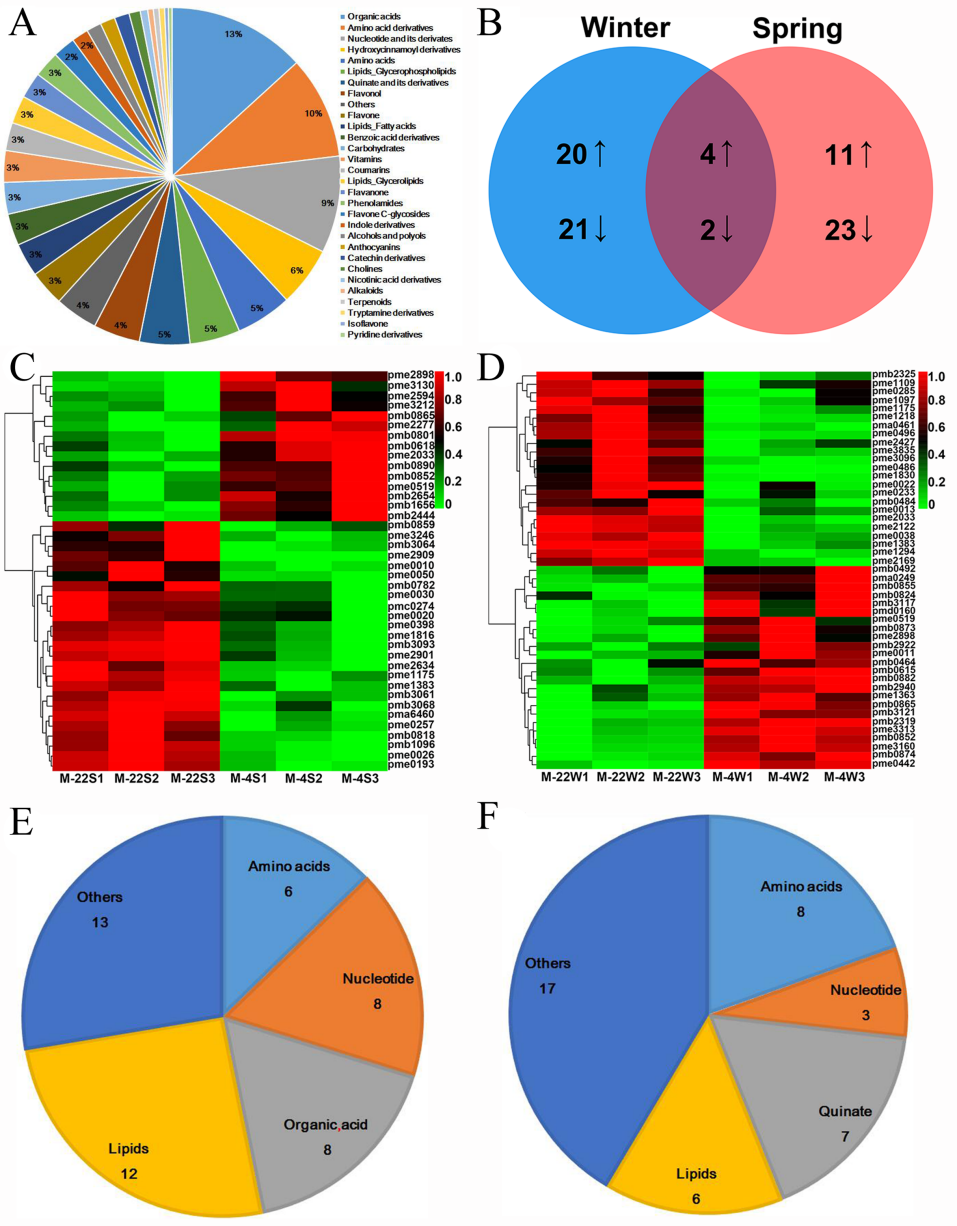
Metabolites identified in the winter and spring *B. napus* ecotypes under cold and normal conditions. (A) Metabolites identified in this study belonged to different classes. (B) Venn diagram of differentially accumulated metabolites between the winter and spring *B. napus* ecotypes after cold stress. (C) Heatmap of differentially accumulated metabolites in the spring *B. napus* ecotype after cold stress. (D) Heatmap of differentially accumulated metabolites in the winter *B. napus* ecotype after cold stress. (E) Differentially accumulated metabolites in the winter *B. napus* ecotype after cold stress mainly belonged to four classes. (F) Differentially accumulated metabolites in the spring *B. napus* ecotype after cold stress mainly belonged to four classes.

To detect the effects of DEGs in response to cold stress at the metabolite level, metabolite profiling of winter and spring *B. napus* ecotypes using LC–MS was performed in this study. In total, 559 metabolites were distributed in 30 classes (Table S11). Among them, the top three classes, namely organic acids (74, 13.2%), amino acid derivatives (55, 9.8%) and nucleotides and their derivatives (52, 9.3%), made up more than 30% of all the metabolites (Fig. 8A). Among these metabolites, 81 were differentially accumulated metabolites (DAMs) using VIP ≥ 1 and *T*-test *P* < 0.05, with 35 increased and 46 decreased after cold stress. In addition, 47 (including 24 increased and 23 decreased) and 40 (including 15 increased and 25 decreased) metabolites were differentially accumulated in winter and spring *B. napus* ecotypes after cold stress (Figs. 8B–8D; Table S12). Among these differentially accumulated metabolites, lipids and amino acids were the most prominent in winter and spring *B. napus* ecotypes, respectively (Figs. 8E and 8F). Four metabolites increased, namely lysoPC 18:2 (lipids glycerophospholipids, pmb0852), lysoPC 18:3 (lipids glycerophospholipids, pmb0865), d-(+)-sucrose (carbohydrates, pme0519) and dihydromyricetin (flavonol, pme2898), and other two metabolites, guanosine (nucleotide and its derivatives, pme1175) and pyridoxine (vitamins, pme1383), decreased in both winter and spring *B. napus* ecotypes (Figs. 8C and 8D; Table S12). Interestingly, L(−)-malic acid (organic acids, pme2033) was increased in spring oilseed rape ecotype, while it was decreased in the winter oilseed rape ecotype (Figs. 8C and 8D; Table S12), suggesting that the metabolites might have critical roles in response to cold stress.

### Combined analysis of gene expression and metabolite data

**Figure 9:**
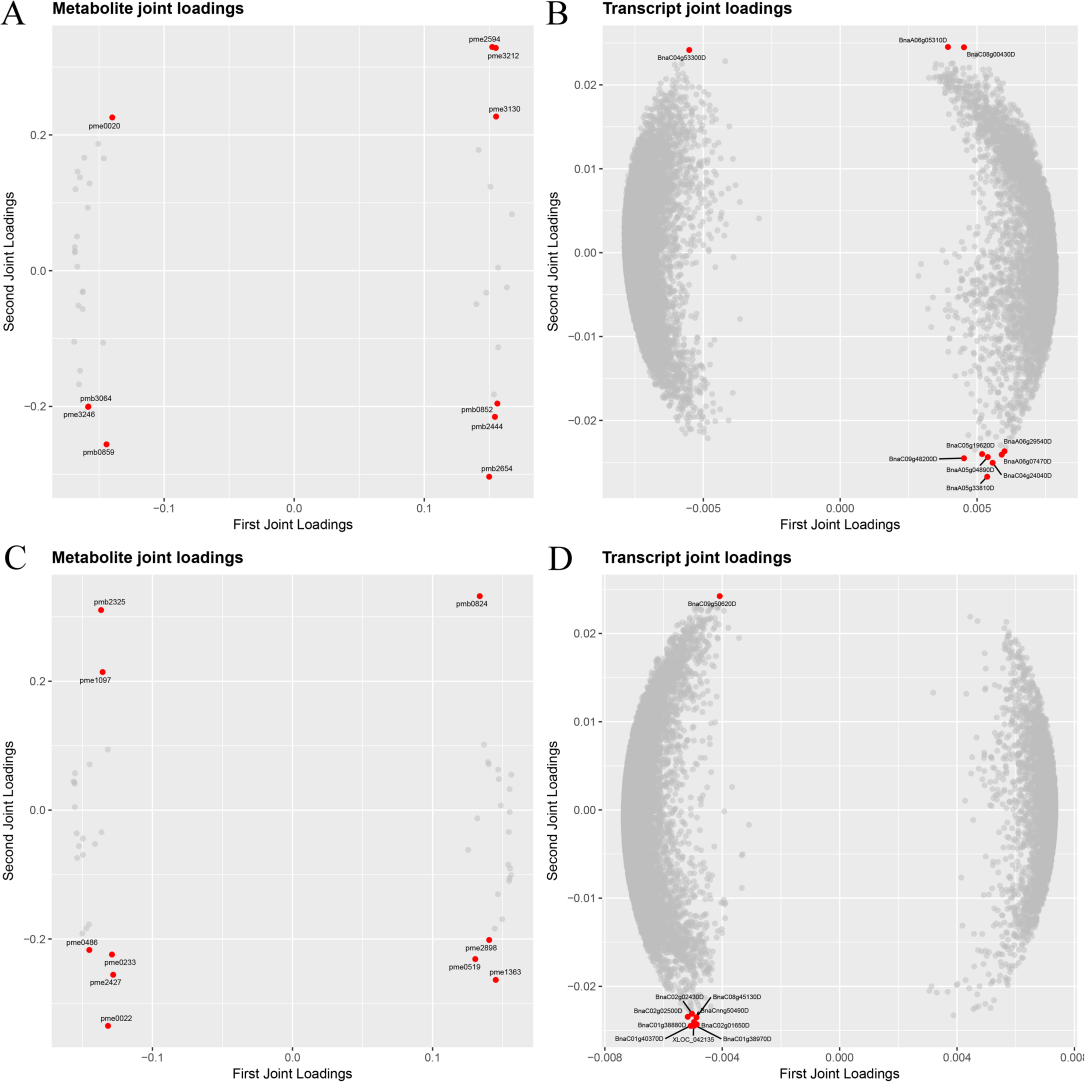
Loading plots of DEGs and DEMs identified in the spring (A and B) and winter (C and D) *B. napus* ecotypes after cold stress. Red circles represent the top 10 genes and 10 metabolites. (A) Metabolite joint loading plot of spring *B. napus* ecotypes. (B) Transcript joint loading plot of spring *B. napus* ecotypes. (C) Metabolite joint loading plot of winter *B. napus* ecotypes. (D) Transcript joint loading plot of winter *B. napus* ecotypes.

To screen key genes and metabolites involved in the response to cold stress, joint data from gene expression and metabolism using pairwise correlations and O2PLS were assessed. A pairwise correlation analysis of all DEGs and DAMs was performed, and the top 50 DEGs and top 50 DAMs are displayed in Fig. 9; Table S13. Two latent variables were identified using O2PLS analysis, with 98.0–98.3% of the total variation in the transcript dataset and 97.4–98.3% of the total variation in the metabolite dataset (Table S14). To visualize the importance of the elements in the two omics, a loading plot of different omics was drawn for all transcriptional and metabolic data (Tables S15 and S16). As shown in Figs. 9A–9C and 9B–9D, the loading coefficient thresholds were higher for the metabolomics dataset than for the transcriptomic dataset, and the top 10 genes and metabolites are represented as a red circular plot (Figs. 9A–9C and 9B–9D). In the winter *B. napus* ecotype, four metabolites (d-(+)-sucrose, nicotinic acid adenine dinucleotide, syringic acid *O*-feruloyl-*O*-hexoside and dihydromyricetin) were increased, and the rest of them (MAG (18:3) isomer2, methylmalonic acid, adenine, l-threonine, *N*-acetyl-l-tyrosine and kynurenic acid) were decreased (Table 2). Interestingly, all ten genes were downregulated, two of which were involved in protein posttranslational modifications, three in the cell, one in amino acid metabolism, one in RNA regulation, one in glycolysis and two encoded unknown proteins (Table 3). In the spring *B. napus* ecotype, four metabolites (Coniferin, 3-*O*-*p*-coumaroyl quinic acid *O*-hexoside, LysoPC 18:1 (2n isomer) and l-phenylalanine) were decreased and six metabolites (MAG (18:3) isomer1, LysoPC 18:2, quercetin 3-*O*-glucoside (isotrifoliin), 4-pyridoxic acid, quercetin 4′-*O*-glucoside (spiraeoside) and anthranilate *O*-hexosyl-*O*-hexoside) were increased (Table 2). Among the top 10 genes, nine were upregulated and one was downregulated with unknown functions. Nine upregulated genes were found to encode MYB111, RAV1, CARBONIC ANHYDRASE 1, FASCICLIN-LIKE ARABINOGALACTAN PROTEIN 8, NICOTIANAMINE SYNTHASE 3, UDP glucosyl and glucoronyl transferases, anthocyanins, anthocyanin 5-aromatic acyltransferase, eukaryotic aspartyl protease family protein and one unknown function protein (Table 3).

**Table 2:** Top 10 metabolites in winter and spring *B. napus* ecotypes based on O2PLS analysis.

Type	ID	VIP	Metabolite name	Class
Spring *B. napus*				
	pme3246	−1.53	Coniferin	Hydroxycinnamoyl derivatives
	pmb3064	−1.26	3-*O*-*p*-coumaroyl quinic acid *O*-hexoside	Quinate and its derivatives
	pmb0859	−0.79	LysoPC 18:1 (2n isomer)	Lipids_Glycerophospholipids
	pme0020	−0.54	l-Phenylalanine	Amino acids
	pmb2444	0.39	MAG (18:3) isomer1	Lipids_Glycerolipids
	pmb0852	0.50	LysoPC 18:2	Lipids_Glycerophospholipids
	pme3212	0.94	Quercetin 3-*O*-glucoside (Isotrifoliin)	Flavonol
	pme2594	1.00	4-Pyridoxic acid	Pyridine derivatives
	pme3130	1.05	Quercetin 4′-*O*-glucoside (Spiraeoside)	Flavonol
	pmb2654	1.79	Anthranilate *O*-hexosyl-*O*-hexoside	Benzoic acid derivatives
Winter *B. napus*				
	pmb2325	−1.38	MAG (18:3) isomer2	Lipids_Glycerolipids
	pme0486	−1.01	Methylmalonic acid	Organic acids
	pme1097	−0.99	Adenine	Nucleotide and its derivates
	pme0022	−0.89	l-Threonine	Amino acids
	pme2427	−0.62	*N*-Acetyl-l-tyrosine	Amino acid derivatives
	pme0233	−0.49	Kynurenic acid	Organic acids
	pme0519	0.43	d-(+)-Sucrose	Carbohydrates
	pme1363	0.60	Nicotinic acid adenine dinucleotide	Nucleotide and its derivates
	pmb0824	0.79	Syringic acid *O*-feruloyl-*O*-hexoside	Benzoic acid derivatives
	pme2898	1.68	Dihydromyricetin	Flavonol

**Table 3:** Top 10 genes in winter and spring *B. napus* ecotypes based on O2PLS analysis.

Type	Gene ID	Log2 fold change	Bin code	Bin name
Spring *B. napus*				
	BnaC04g53300D	−1.01	–	–
	BnaA06g29540D	1.03	27.3.25	RNA.regulation of transcription
	BnaA06g07470D	1.07	27.3.3	RNA.regulation of transcription
	BnaA05g33810D	1.07	8.3	TCA/org transformation
	BnaA05g04890D	1.15	10.5.1.1	cell wall.cell wall proteins
	BnaC09g48200D	1.16	27.3.67	RNA.regulation of transcription
	BnaC05g19620D	1.31	26.2	misc.UDP glucosyl and glucoronyl transferases
	BnaC04g24040D	1.32	–	–
	BnaC08g00430D	1.45	16.8.1.21	secondary metabolism.flavonoids.anthocyanins
	BnaA06g05310D	1.58	15.2	metal handling.binding
Winter *B. napus*				
	BnaC09g50620D	−1.03	29.4	protein.postranslational modification
	BnaC01g40370D	−1.00	29.4	protein.postranslational modification
	BnaCnng50490D	−1.42	31.1	cell.organization
	BnaC02g02500D	−1.19	31.4	cell.vesicle transport
	BnaC01g38970D	−1.27	31.4	cell.vesicle transport
	BnaC01g38880D	−1.59	13.1.4.5.1	amino acid metabolism
	BnaC02g01650D	−1.17	27.3.73	RNA.regulation of transcription
	BnaC08g45130D	−1.04	4.1.6	glycolysis.cytosolic branch
	BnaC02g02430D	−1.32	–	–
	XLOC_042135	−1.15	–	–

## Discussion

As one major adverse environmental factor, low temperature decreases crop production worldwide and has become a great concern for crop breeders (Khodakovskaya et al., 2006; Chinnusamy, Zhu & Zhu, 2007). Plants have evolved fine regulatory mechanisms in response to low temperature stress, including changes in gene expression and metabolites (Maruyama et al., 2014). In this study, alterations of genes and metabolites after cold stress in both winter and spring *B. napus* ecotypes were detected using RNA-Seq and LC-MS analysis, respectively.

### Roles of lipid metabolism in response to cold stress

The plasma membrane performs crucial roles in response to low temperature stress, and lipids are the major component of the plasma membrane (D’Angeli & Altamura, 2016). Genes involved in lipid metabolism were differentially expressed after cold stress, as shown by the enrichment of fatty acid biosynthesis (ko00061), fatty acid elongation (ko00062), alpha-linolenic acid metabolism (ko00592), steroid biosynthesis (ko00100), glycerolipid metabolism (ko00561) and glycerophospholipid metabolism (ko00564) pathways (Fig. 6; Table S7). Key genes encoding proteins, including sterol methyltransferase 3 (SMT3), sterol 1 (STE1), lipase 1 (LIP1), phospholipase D alpha 1 (PLDALPHA1), diacylglycerol kinase1 (DGK1), glycerol-3-phosphate acyltransferase 4 (GPAT4) and lysophosphatidyl acyltransferase 2 (LPAT2), were detected as DEGs after cold stress. These genes were involved in the cold response by altering the phospholipid and lipid composition (Li et al., 2004; Testerink & Munnik, 2005; Rodriguez-Vargas et al., 2007). In addition, 19 phospholipid signaling genes were cold-regulated, most of which (15 genes) were upregulated in both winter and spring *B. napus* ecotypes. These genes included three PLD2s, two INOSITOL (1, 4 and 5) P3 5-PHOSPHATASE IIs (IP5P2), two PLC-like phosphodiesterase superfamily protein genes (plcA), four DGK1s, three phospholipase C1s (PLC1), three DGK2s and two inositol monophosphatase family protein genes (SAL3). Taken together, these differentially expressed cold-regulated genes indicated that phospholipid second messengers play critical roles in response to cold signaling. Diacylglycerol (DAG) is generated from phosphatidylinositol 4, 5-bisphosphate by PLCs and converted to phosphatidic acid (PA) by DGKs (Munnik, 2001). PLDs are used to catalyze the transformation of phosphatidylcholine and phosphatidylethanolamine to generate PA. An increasing number of reports suggest that PA plays key roles in cold signaling (Ruelland et al., 2002; Welti et al., 2002; Li et al., 2004). In the metabolism analysis, lysoPC 18:2 (pmb0852) and lysoPC 18:3 (2n isomer, pmb0865) were accumulated in both winter and spring *B. napus* ecotypes. Interestingly, three glycerolipids, namely MAG (18:2, pmb0890), MAG (18:3) isomer1 (pmb2444) and MAG (18:3) isomer4 (pmb1656), were significantly accumulated only in the spring *B. napus* ecotype, while eight glycerophospholipids, including lysoPC 16:0 (pmb0855), lysoPE 16:0 (pmb3117), lysoPE 16:0 (2n isomer, pmd0160), lysoPC 15:0 (pmb2319), lysoPC 18:1 (pmb0882), lysoPC 18:2 (2n isomer, pmb0873), lysoPE 18:1 (pmb3121) and lysoPE 18:2 (2n isomer, pmb0874), were significantly accumulated only in the winter *B. napus* ecotype. However, lysoPC 18:1 (2n isomer, pmb0859) was decreased in the spring *B. napus* ecotype, and MAG (18:3) isomer2 (pmb2325) and 14, 15-dehydrocrepenynic acid (pma0461) were decreased in the winter *B. napus* ecotype after cold stress. These findings suggest that lipid metabolism may have crucial functions in response to cold stress.

### ABA signal transduction components have critical roles in response to cold stress

Increasing evidence has revealed the types of mechanisms involved in cold stress response. As an important stress phytohormone, ABA participates in regulating diverse abiotic stresses. COR genes are precisely regulated by both ABA-dependent and -independent pathways. Hormone homeostasis may be altered under cold stress compared with normal conditions (Lee, Henderson & Zhu, 2005). ABA levels are increased after cold stress in potato (Chen, Li & Brenner, 1983). Plant freezing tolerance can be enhanced by exogenous application of ABA at a normal temperature, while the functions of ABA in the cold regulation process remain elusive.

To explore the involvement of ABA in the cold stress response in *B. napus*, we detected the expression patterns of ABA biosynthesis and the ABA signaling process in two *B. napus* ecotypes after cold stress. In this study, 27 genes encoding the key rate-limiting enzymes of ABA biosynthesis, including nine-*cis*-epoxycarotenoid dioxygenase 3 (NCED3), NCED4, NCED5, NCED9, ABA DEFICIENT 1 (ABA1), ABA2, abscisic aldehyde oxidase 3 (AAO3), aldehyde oxidase 1 (AAO1), AAO2 and carotenoid cleavage dioxygenase 1 (CCD1), were observed in the two *B. napus* ecotypes after cold stress (Table S5). Among them, 16 genes, 10 down- and 6 upregulated genes were commonly detected in the two *B. napus* ecotypes after cold stress, while five and six ABA biosynthesis genes were specific to the winter and spring *B. napus* ecotype after cold stress (Table S5), respectively. These data are consistent with previous studies conducted in Arabidopsis (Lang et al., 1994; Park et al., 2008) and *Populus euphratica* (Chen et al., 2014), indicating that ABA biosynthesis is regulated by cold. In ABA signal transduction, many ABA-induced genes, encoding ABA INSENSITIVE 1 (ABI1), ABI5, ABA-responsive element binding protein 3 (AREB3), abscisic acid responsive element-binding factor 1 (ABF1), ABF2, ABF3, highly ABA-induced PP2C gene 1 (HAI1), HAI2, HAI3, OPEN STOMATA 1 (OST1), PYR1-like 4 (PYL4), PYL6, PYL7, PYL10, regulatory component of ABA receptor 1 (RCAR1), RCAR3, SNF1-related protein kinase 2.2 (SNRK2.2), SNRK2.5, SNRK2.7 and SNRK2.10, were differentially expressed in winter and/or spring *B. napus* ecotype(s) after cold stress (Table S5). ABF1, which binds to the ABA-responsive element in ABA-responsive promoters, was continually induced by cold in Arabidopsis (Lee, Henderson & Zhu, 2005). One copy of *BnABF1* and two copies of *BnABF3*s were commonly induced in both winter and spring *B. napus* ecotypes, while two copies of *BnABF2*s were downregulated and only observed in the winter *B. napus* ecotype after cold stress (Table S5). Regardless, our data indicate that ABA functions as a crucial mediator of the cold stress response in *B. napus*.

### Alterations in secondary metabolism in response to cold stress

Genes involved in secondary metabolism, including flavonoid biosynthesis, flavone and flavonol biosynthesis, glucosinolate biosynthesis, monobactam biosynthesis, phenylpropanoid biosynthesis and stilbenoid, diarylheptanoid and gingerol biosynthesis, may play critical roles in response to cold stress (Fig. 6; Table S7). In the flavonoid biosynthesis process, two copies of *flavonol synthase 1*s (*FLS1*s, *BnaA10g22800D* and *BnaC09g47360D*), one *FLS3* (*BnaC02g42580D*), two *TRANSPARENT TESTA 4*s (*TT4*, *BnaC02g05070D* and *BnaC09g43250D*), two *TT5*s (*BnaA09g34840D* and *BnaC08g26020D*) and one *dihydroflavonol 4-reductase* (*DFR*, *BnaC09g17150D*), were upregulated in both winter and spring *B. napus* ecotypes. Interestingly, one *FLS1* (*BnaCnng61390D*), one *DFR* (*BnaA09g15710D*), two *leucoanthocyanidin dioxygenase*s (*LDOX*, *BnaC01g14310D* and *BnaC07g37670D*), two *TT5*s (*BnaA07g37900D* and *BnaC08g26010D*) and three *TT4*s (*BnaA03g04590D*, *BnaA10g19670D* and *BnaC03g06120D*) were only upregulated in the spring *B. napus* ecotype. Unfortunately, no metabolite changes were detected in this process. *BnaA02g03370D* and *BnaC02g06990D* encoding UDP-glucosyl transferase 78D2 (UGT78D2) involved in flavone and flavonol biosynthesis were also commonly upregulated in the two *B. napus* ecotypes. Quercetin 4′-*O*-glucoside (spiraeoside) and quercetin 3-*O*-glucoside (isotrifoliin) belonging to flavonol were only increased in the spring *B. napus* ecotype, while selgin 5-*O*-hexoside (flavone) and hesperetin C-hexosyl-*O*-hexosyl-*O*-hexoside (flavone C-glycosides) were only increased in the winter *B. napus* ecotype after cold stress (Table S10). In total, 17 genes involved in monobactam biosynthesis were downregulated in both winter and spring *B. napus* ecotypes. Genes encoding ATP-SULFURYLASE 3 (APS3), APS4, aspartate kinase 1 (AK-LYS1), dihydrodipicolinate synthase 1 (DHDPS1), DHDPS2 and semialdehyde dehydrogenase family protein were detected and participated in S-assimilation and amino acid metabolism processes. Genes that were upregulated in the spring *B. napus* ecotype after cold stress included seven copies of *cytochrome P450, family 71, subfamily B, polypeptide 17*s (*CYP71B17*s), one *CYP76G1*, two *CYP81F4*s, one *CYP81D8* and three *CYP91A2*s. By contrast, chlorogenic acid (3-*O*-caffeoylquinic acid, pme0398) was decreased in the spring *B. napus* ecotype after cold stress. This metabolite can function in the oxidative stress response. Commonly upregulated in both winter and spring *B. napus* ecotypes after cold stress were lignin biosynthesis genes encoding PHE ammonia lyase 1 (PAL1), cinnamate-4-hydroxylase (C4H), cinnamyl alcohol dehydrogenase 5 (CAD5), *O*-methyltransferase 1 (OMT1), ferulic acid 5-hydroxylase 1 (FAH1), caffeoyl-CoA 3-*O*-methyltransferase (CCOAMT) and hydroxycinnamoyl-CoA shikimate/quinate hydroxycinnamoyl transferase (HCT). Coniferin (pme3246), chlorogenic acid (3-*O*-caffeoylquinic acid, pme0398), l-(−)-tyrosine (pme0030) and l-phenylalanine (pme0020) were decreased in the spring *B. napus* ecotype after cold stress.

### Signal transduction in response to cold stress

The signal transduction pathway makes great contributions in response to various environmental stresses, including cold stress (Janska et al., 2010; D’Angeli & Altamura, 2016; Tanou et al., 2017). As an important messenger, Ca^2+^ plays a crucial role in stress signaling (Ye et al., 2013). The Ca^2+^ level increases rapidly under low temperature stress and activates various signaling pathways in response to cold stress and cold-induced gene regulatory processes (Monroy & Dhindsa, 1995; Dodd, Kudla & Sanders, 2010; Reddy et al., 2011; Shi, Ding & Yang, 2015). Calcium signals are sensed by three major family proteins in higher plants: calmodulins (CaMs), calcium-dependent protein kinases (CDPK) and calcineurin B-like (CBL) proteins (Sheen, 1996; Luan, 2009; Boudsocq & Sheen, 2013; Miura & Furumoto, 2013). The MAPKs cascade and receptor-like protein kinases (RLKs) also contribute in the perception of signals in the external environment and have important roles in environmental stress responses (Morris & Walker, 2003; Lehti-Shiu et al., 2009). The calmodulin-like *OsMSR2* gene is significantly induced by low temperature, and its over-expression in Arabidopsis enhances the salt and drought tolerance ability of the plants (Xu et al., 2011). Five calmodulin genes, two *CDPK* genes and one *CBL* gene in *Camellia sinensis* were identified to take part in signal transduction after cold stress using RNA-Seq analysis (Wang et al., 2013b). To determine whether these gene families were involved in cold response in *B. napus*, we explored these genes in the list of DEGs. The results showed that 135 genes, including 44 *MAPK* cascade genes, 35 *CDPK*s, 38 *CaM*s, 16 *CIPK*s and 2 Ca^2+^-ATPase genes, were differentially expressed, with 68 upregulated and 67 downregulated in winter *B. napus* ecotype after cold stress. In the spring *B. napus* ecotype, 117 genes, including 38 *MAPK* cascade genes, 44 *CDPK*s, 19 *CaM*s, 15 *CIPK*s and 1 Ca^2+^-ATPase genes, were differentially expressed, with 34 upregulated and 83 downregulated after cold stress (Table S17). Among them, 54 genes (including 25 *MAPK* cascade genes, 26 *CDPK*s, 14 *CaM*s, 13 *CIPK*s and 1 Ca^2+^-ATPase gene) were commonly detected, with 27 upregulated and 52 downregulated in both winter and spring *B. napus* ecotypes. Interestingly, two genes, encoding CBL1 (*BnaA01g08510D*) and ACA2 (*BnaA08g15730D*), showed opposite expression patterns between the two *B. napus* ecotypes after cold stress. These two genes were downregulated in the winter *B. napus* ecotype but upregulated in the spring *B. napus* ecotype after cold stress (Table S17). Our results provide further evidence supporting the key functions of Ca^2+^ binding proteins and the MAPK pathway in response to cold stress in *B. napus*.

### TFs in response to cold stress

Transcription factors play key roles in various biological processes, including diverse stress responses (including cold stress), via binding to cis-acting elements of target genes (Singh, Foley & Onate-Sanchez, 2002; Yamaguchi-Shinozaki & Shinozaki, 2006; Chinnusamy, Zhu & Sunkar, 2010; Lindemose et al., 2013). In this study, various kinds of TF genes were differentially expressed after cold stress (Fig. 3; Table S5), and many of them are involved in the cold stress response in plants (Jakoby et al., 2002; Toledo-Ortiz, Huq & Quail, 2003; Mukhopadhyay, Vij & Tyagi, 2004; Hu et al., 2008; Rushton et al., 2010; Chen et al., 2014; Jiang et al., 2017). Many cold-related (COR) genes contain dehydration-responsive elements/C-repeat elements (DRE/CRT, A/GCCGAC) and myeloblastosis (MYB, C/TAACNA/G) in their promoters and are regulated by AP2/ERF and MYB TFs (Stockinger, Gilmour & Thomashow, 1997; Yamaguchi-Shinozaki & Shinozaki, 2005). CBF TFs, including DREB1A/CBF3, DREB1B/CBF1 and DREB1C/CBF2, belong to the AP2/ERF family, which is divided into the AP2, RAV, ERF and DREB subfamilies (Yamasaki & Randall, 2016; Vazquez-Hernandez et al., 2017). Additionally, AP2/ERF play crucial regulatory functions in response to cold stress, especially DREB proteins, which regulate ABA-independent genes involved in low temperature stress in *A. thaliana* (Du et al., 2016). DREB2B also enhances tolerance to various abiotic stresses (including cold stress) by increasing proline levels in both yeast and tobacco (Li et al., 2014). In our study, 211 *BnAP2/ERF* genes were differentially expressed, and two *BnCBF1*s, five *BnCBF2*s (*BnaC03g71900D* was only upregulated in the spring ecotype) and two *BnCBF4*s (*BnaA10g07630D* was only upregulated in the spring ecotype) were strongly upregulated in both winter and spring *B. napus* ecotypes after cold stress (Table S5). By contrast, no *BnCBF3*s was found to be upregulated in the two *B. napus* ecotypes after cold stress (Table S5). Two copies of *BnDREB2B*s, *BnaA05g27930D* were overrepresented in both ecotypes, and *BnaAnng23490D* was only upregulated in the winter *B. napus* ecotype after cold stress (Table S5). *BnERF7*s and *BnERF38*s were overrepresented, while *BnERF1*s, *2*s, *5*s, *6*s and *13*s were downregulated after cold stress in the two *B. napus* ecotypes (Table S5).

Several COR genes are not directly regulated by the CBF/DREB family (Fowler & Thomashow, 2002; Kreps et al., 2002). WRKY and NAC TFs also play key roles in response to cold stress (Hu et al., 2008; Yokotani et al., 2013; Yang et al., 2015; Zhuo et al., 2018). An involvement of the WRKY gene family in the cold stress response has been suggested in Arabidopsis (Lee, Henderson & Zhu, 2005), rice (Ramamoorthy et al., 2008), soybean (Zhou et al., 2008), *Vitis vinifera* (Wang et al., 2014), cucumber (Zhang et al., 2016) and cassava (Wei et al., 2016). In our study, 150 (128 in the winter and 115 in the spring ecotype) genes were differentially regulated after cold stress, with 47 up- and 103 downregulated. Among them, 26 up- and 46 downregulated were detected in both winter and spring *B. napus* ecotypes after cold stress. Key members, such as *BnWRKY8*s, *23*s, *28*s, *31*s, *42*s, *48*s and *123*s, were upregulated, while *BnWRKY7*s, *51*s, *70*s and *74*s were downregulated after cold stress in winter and/or spring *B. napus* ecotypes. Key members of *NAC* genes involved in plant cold responsiveness have been reported, such as *TaNAC2*, *OsNAC5* and *SsNAC23* (Hu et al., 2008; Song et al., 2011). Thirty-two and twenty-eight NAC genes were upregulated after cold stress in the winter and spring *B. napus* ecotypes, respectively, and nine of them were common to the two ecotypes. Many other kinds of TFs, such as bHLH, C2H2, MYB, MYC, MADS and b-ZIP families, were also detected as DEGs after cold stress (Table S5), suggesting that these TFs are also involved in the low temperature regulatory process (Chinnusamy et al., 2003; Yang, Dai & Zhang, 2012; Peng et al., 2013). These up-and downregulated TFs suggest very complex mechanisms of low temperature regulation.

## Conclusions

In this study, a total of 25,460 DEGs and 41 differentially expressed metabolites (DEMs) in spring oilseed ecotype and 28,512 DEGs and 47 DEMs in winter oilseed ecotype were identified by transcriptome and metabolome analyses after cold stress. In addition, more specific genes and metabolites were detected in winter ecotypes. The combined data indicate that lipid, ABA, secondary metabolism, signal transduction and TFs could be involved in the complex regulation in the spring and winter ecotypes in response to cold stress. This study provides new evidence and insights into how different ecotypes of *B. napus* respond to cold stress from metabolomics and transcriptomics levels.

## Supplemental Information

10.7717/peerj.8704/supp-1 Supplemental Information 1 Pearson coefficients among three biological replicates.

10.7717/peerj.8704/supp-2 Supplemental Information 2 Gene Ontology (GO) classification of DEGs identified in the winter and spring *B. napus* ecotypes after cold stress. (A) Common DEGs identified in the winter and spring *B. napus* ecotypes after cold stress. (B) DEGs identified only in the spring *B. napus* ecotype after cold stress. (C) DEGs identified only in the winter *B. napus* ecotype after cold stress.

10.7717/peerj.8704/supp-3 Supplemental Information 3 Primers used for qRT-PCR in this study.

10.7717/peerj.8704/supp-4 Supplemental Information 4 Overview of transcriptome sequencing and mapping to the *B. napus* reference genome.

10.7717/peerj.8704/supp-5 Supplemental Information 5 Overview of all genes detected from 12 libraries by RNA-sequencing.

10.7717/peerj.8704/supp-6 Supplemental Information 6 Differentially expressed genes identified in the two *B. napus* ecotypes after cold stress.

10.7717/peerj.8704/supp-7 Supplemental Information 7 Statistical analysis of DEGs belonging to different transcription factor families in winter and spring *B. napus* ecotypes after cold stress.

10.7717/peerj.8704/supp-8 Supplemental Information 8 DEGs belonging to hormone biosynthesis and signal transduction process in winter and spring *B. napus* ecotypes after cold stress.

10.7717/peerj.8704/supp-9 Supplemental Information 9 Identification of DEGs related to temperature stress response in winter and spring *B. napus* ecotypes after cold stress.

10.7717/peerj.8704/supp-10 Supplemental Information 10 Significantly enriched pathways of DEGs in winter and spring *B. napus* ecotypes after cold stress.

10.7717/peerj.8704/supp-11 Supplemental Information 11 MapMan Bins of DEGs enriched in different metabolic processes in spring *B. napus* ecotype after cold stress.

10.7717/peerj.8704/supp-12 Supplemental Information 12 MapMan Bins of DEGs enriched in different metabolic processes in winter *B. napus* ecotype after cold stress.

10.7717/peerj.8704/supp-13 Supplemental Information 13 Metabolites identified in winter and spring *B. napus* ecotypes after cold stress using LC-MS analysis.

10.7717/peerj.8704/supp-14 Supplemental Information 14 Differentially accumulated metabolites identified in the two *B. napus* ecotypes after cold stress.

10.7717/peerj.8704/supp-15 Supplemental Information 15 Correlation coefficents of top 50 DEGs and DEMs based on pairwise correlations analysis.

10.7717/peerj.8704/supp-16 Supplemental Information 16 Variation of transcript and metabolome.

10.7717/peerj.8704/supp-17 Supplemental Information 17 Loading coefficients of metabolites used for loading plot based on O2PLS analysis.

10.7717/peerj.8704/supp-18 Supplemental Information 18 Loading coefficients of genes used for loading plot based on O2PLS analysis.

10.7717/peerj.8704/supp-19 Supplemental Information 19 DEGs related to signal transduction process identified in winter and spring *B. napus* ecotypes after cold stress.

## Additional Information and Declarations

### Data Availability

The following information was supplied regarding data availability: Data is available at the NCBI: PRJNA530291.
