# Supplementary figures and images for: Characterization of cold stress responses in different rapeseed ecotypes based on metabolomics and transcriptomics analyses
Source: PeerJ. 2020 Mar 31;8:e8704. doi: 10.7717/peerj.8704 (PMC7120054; doi:10.7717/peerj.8704)

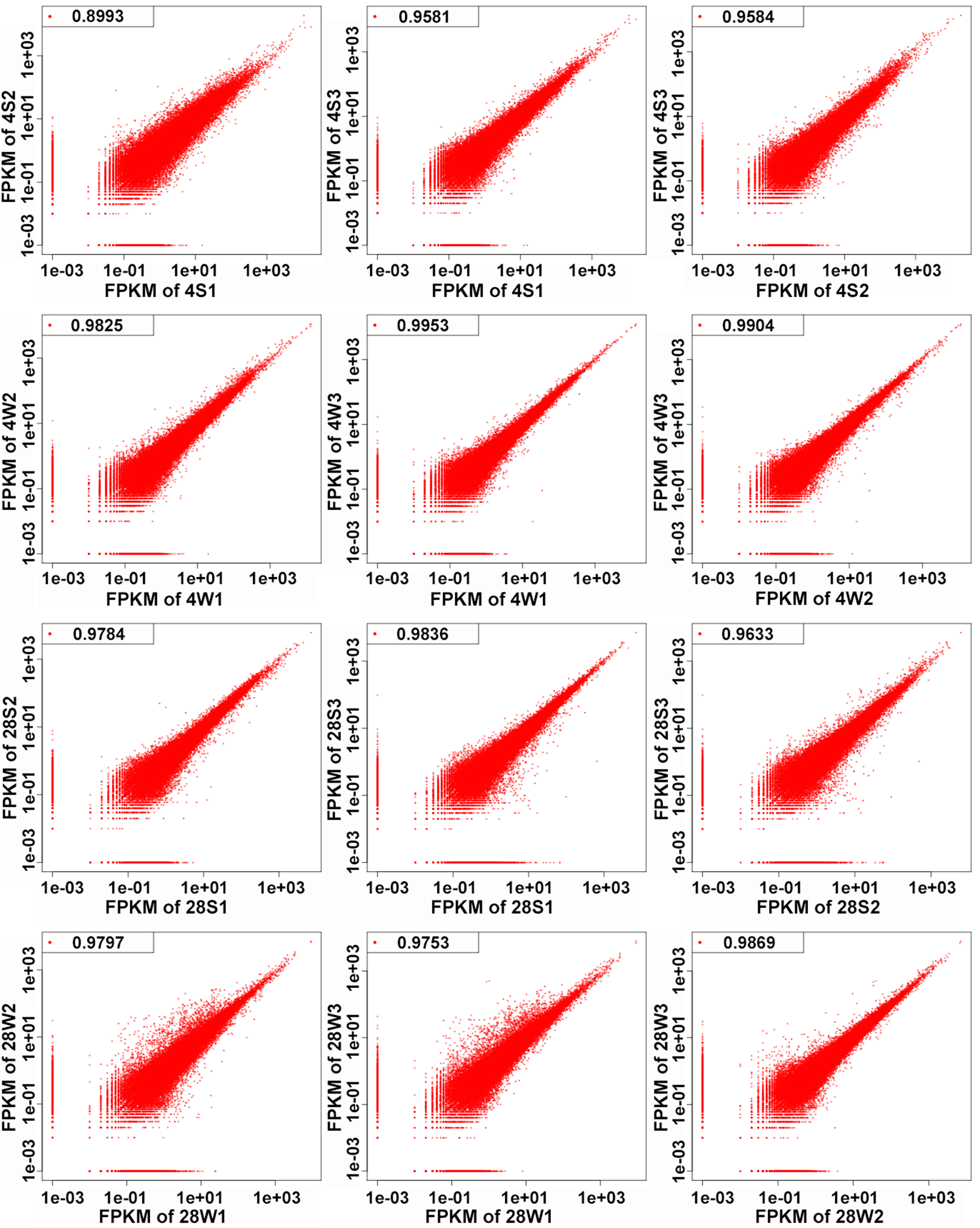

Supplement: Supplemental Information 1 [file peerj-08-8704-s001.png]

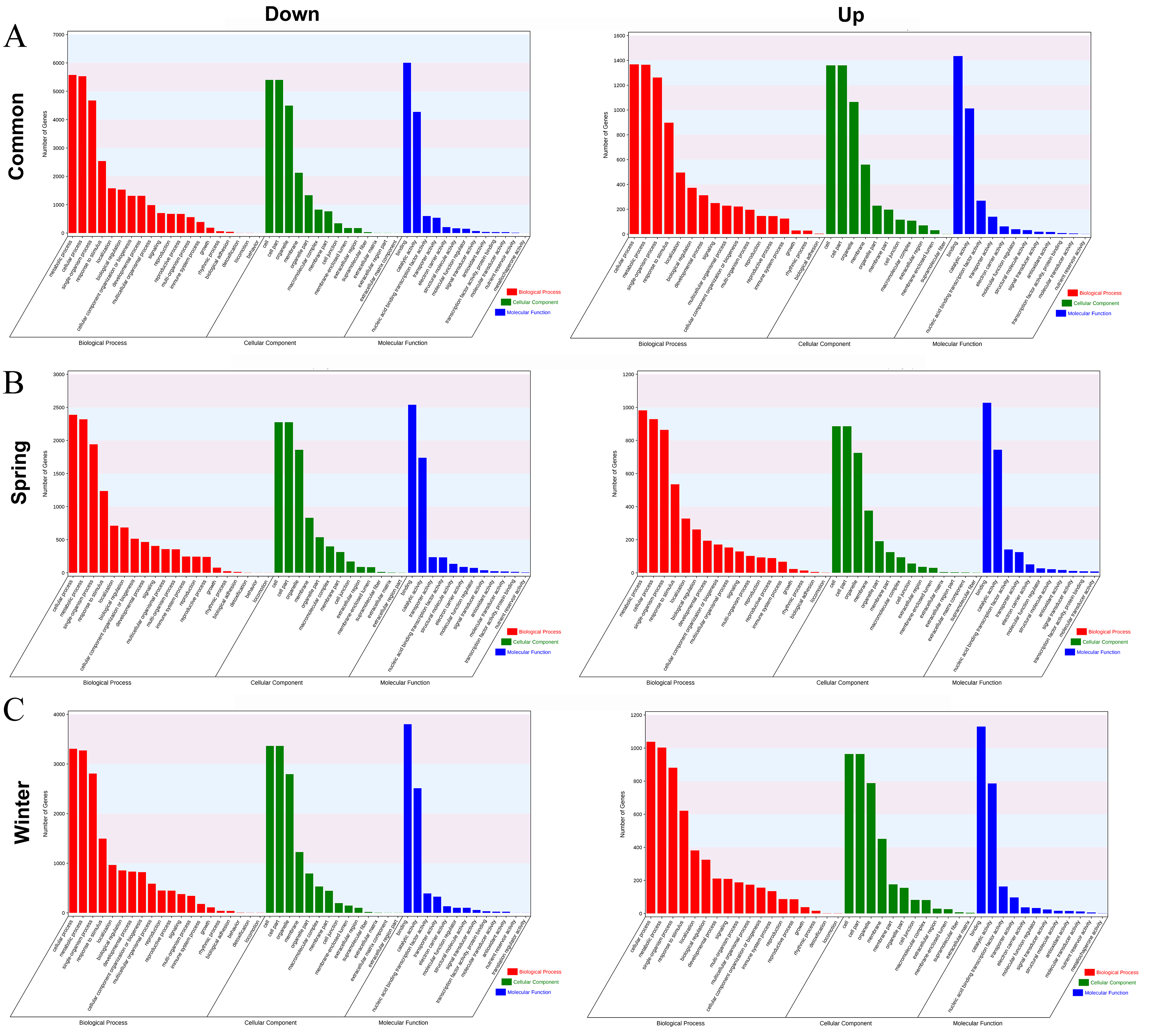

Supplement: Supplemental Information 2 — (A) Common DEGs identified in the winter and spring B. napus ecotypes after cold stress. (B) DEGs identified only in the spring B. napus ecotype after cold stress. (C) DEGs identified only in the winter B. napus ecotype after cold stress. [file peerj-08-8704-s002.png]
